# Sexually selected UV signals in the tropical ornate jumping spider, *Cosmophasis umbratica* may incur costs from predation

**DOI:** 10.1002/ece3.1419

**Published:** 2015-01-28

**Authors:** Matthew W Bulbert, James C O'Hanlon, Shane Zappettini, Shichang Zhang, Daiqin Li

**Affiliations:** 1 Behavioural Ecology Group, Department of Biological Sciences, Macquarie UniversityNorth Ryde, New South Wales, Australia; 2 Center for the Integrated Study of Animal Behavior, Program in Cognitive Science, Indiana UniversityBloomington, Indiana; 3 Behaviour Ecology and Sociobiology Lab, Department of Biological Sciences, National University of SingaporeSingapore, Singapore; 4 Centre for Behavioural Ecology and Evolution, College of Life Sciences, Hubei UniversityWuhan, 430062, Hubei, China

**Keywords:** Eavesdropping, *Portia*, predation, trade-offs, UV

## Abstract

Sexually selected ornaments and signals are costly to maintain if they are maladaptive in nonreproductive contexts. The jumping spider *Cosmophasis umbratica* exhibits distinct sexual dichromatism with males displaying elaborate UV body markings that signal male quality. Female *C. umbratica* respond favorably to UV-reflecting males and ignore males that have their UV masked. However, *Portia labiata*, a UV-sensitive spider-eating specialist and a natural predator of *C. umbratica*, is known to use UV reflectance as a cue when hunting prey. We investigated the cost of these UV signals in *C. umbratica* in terms of their predation risk. Under experimental conditions, three choice scenarios were presented to *P. labiata* individuals. Choices by *P. labiata* were made between male *C. umbratica* with and without the UV signal; a UV-reflecting male and non-UV-reflecting female; and a UV-masked male and female. The presence and absence of UV signals was manipulated using an optical filter. *Portia labiata* exhibited a strong bias toward UV+ individuals. These results suggest the sexually selected trait of UV reflectance increases the visibility of males to UV-sensitive predators. The extent of this male-specific UV signal then is potentially moderated by predation pressure. Interestingly though, *P. labiata* still preferred males to females irrespective of whether UV reflectance was present or not. This suggests *P. labiata* can switch cues when conditions to detect UV reflectance are not optimal.

## Introduction

Male ornate colorations, elaborate adornments, and complex displays are often implicated in mate choice (Andersson 1994). Typically, they are honest signals of mate quality and are thus used as criteria by females to select a suitable mate. Of course, for a system of mate selection to persist, there must be a process that maintains variability between the males. Two commonly proposed mechanisms include condition dependence in which the traits vary according to the nutritional history of the animal and/or through selective pressures that impose a cost on displaying conspicuously such as predation, that is, handicap principle (Kuijper et al. 2012).

Variation between conspecifics in their sexually selected colorful traits can potentially reflect a trade-off between conspicuousness and concealment (Andersson 1994; Stuart-Fox and Ord 2004). The male with the brightest or most intense hue (the most conspicuous) may have the best mating success but at the cost of a greater risk of increased detection by predators (Stuart-Fox et al. 2003; Husak et al. 2006). Under such circumstances, intense predatory pressure is expected to drive selection for more cryptic coloration, preventing runaway selection for greater conspicuousness. This theory has been extensively supported for displays in the visible spectrum (e.g., (Stuart-Fox and Ord 2004; Kodric-Brown 1985)), but such studies have seldom considered the role of short-wave colorations, that is, ultraviolet (UV) markings.

Jumping spiders are an enigmatic group of predators and as a group they share a remarkable array of predatory modes (Jackson and Pollard 1996). They partake in elaborate mating and contest rituals that use both dynamic and static signals (Lim and Li 2004; Elias et al. 2006). For many species, this involves the display of colors that are sex specific, with possibly the most notable being species of peacock spiders (Girard and Endler 2014). To match their colorful displays, jumping spiders have an equally impressive visual system (Harland et al. 2012) that allows them to discriminate and recognize objects (Harland and Jackson 2000) and colors (Nakamura and Yamashita 2000; Taylor et al. 2014) across visible to short-wave spectra (Hu et al. 2012). Jumping spiders, in general, use three primary colors, which are blue, green, and UV that peaks between 330 and 380 nm. The sensitivity of jumping spiders to short-wavelength spectra has been shown to be extremely important in both intra- and interspecific interactions.

**Figure 1:**
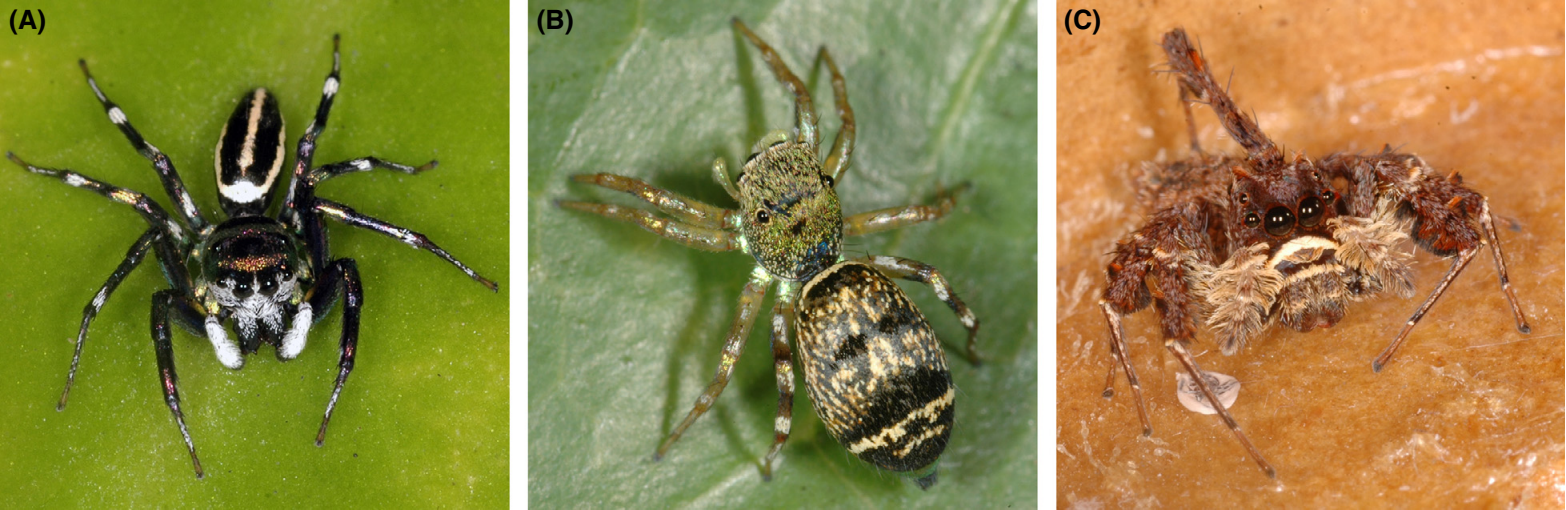
The color variation between study animals; (A) Male *Cosmophasis umbratica*; (B) Female *C. umbratica*; and (C) Male *Portia labiata*.

The tropical ornate jumping spider, *Cosmophasis umbratica*, exhibits distinct sexual dichromatism (Fig.1) (Lim and Li 2006). All body parts used by males during courtship rituals reflect UV. These include the UV-green iridescence on the carapace and abdominal markings and the male's UV-white facial markings and pedipalps (sperm transfer organs) (Fig.1A). The UV-green iridescence, which characterizes the male abdomen, is known to be condition dependent – varying with nutritional intake and age (Lim and Li 2007). The UV-reflecting carapace in contrast varies between males, independent of body condition (Lim and Li 2006). Unlike the males, the juveniles and females (Fig.1B) lack the UV-green iridescence (Lim and Li 2006), which infers the male UV reflectance is a sexually selected trait. Indeed, females spend more time to observing UV-reflecting males than males without UV signals (Lim et al. 2007a) and will not engage in courtship rituals with males where the UV is masked (Lim et al. 2007b). The observed variation in the intensity of UV reflectance between males has been suggested to be an honest signal of male quality (Lim and Li 2013). For instance, the male with the greatest distance between the UV and VIS components of its UV-green iridescence is more likely to win a male-male contest. Presumably then, males with intense UV-iridescence potentially have greater mating success. The extent of male UV reflectance, as stated, varies between males, but the processes that maintain this variation have yet to be established.

A potential cost of conspicuous coloration is that predators may eavesdrop on the signal (Bernal et al. 2006). The white-mustached jumping spider, *Portia labiata*, is a spider-hunting specialist and a natural predator of *C. umbratica*. *Portia labiata*, like *C. umbratica* and many other jumping spiders, can detect UV reflectance (Hu et al. 2012). Unlike *C. umbratica,* neither sex of *P. labiata* is colorful (Fig.1C) (Jackson and Hallas 1986), hence their ability to discern UV is unlikely to be maintained by sexual selection. Instead, *P. labiata's* sensitivity to UV appears to be important for foraging. Both *P. labiata* and *C. umbratica* forage among the leaves and stems of low-lying vegetation (Jackson and Hallas 1986; Lim and Li 2007). Such environments are UV absorbing, so UV reflectance will stand out from the background. They have been shown to cue in on UV-reflecting silk when locating web-building spiders (Li and Lim 2005; Zou et al. 2011). Silk reflects no color in the visible spectrum, and so its detection is unambiguously via its capacity to reflect UV. This suggests UV sensitivity in *P. labiata* has a functional role in locating prey items and hence may enhance their capacity to locate prey that express UV reflectance. Due to this UV sensitivity, we predict *P. labiata* will preferentially attack UV-reflecting male *C. umbratica* over males that have their UV masked. Additionally, if UV reflection is an important cue for *P. labiata*, we would expect females that are non-UV-reflecting to be ignored in the presence of UV-reflecting males. If established, it would provide initial support for the idea that opposing selective pressures may shape UV signals in male *C. umbratica*.

## Materials and Methods

Details for animal collection and maintenance are as described in (Tovée 1995; Lim et al. 2007b). *Portia labiata* were kept under the same conditions but were not fed for 2 weeks prior to the experiment.

### Choice experiment

For each experiment, *P. labiata* individuals were given a task to choose between two *C. umbratica* individuals. The individuals were presented simultaneously and varied in UV reflection, size, and/or sex. Two choice chambers made of Plexiglas (L × B × H = 7.6 × 2.5 × 2.5 cm) were placed side-by-side directly in front of the test subject. They were placed at the junction of a 10^°^ upward sloping wooden Y-shaped frame as described in (Li and Lim 2005). This frame has a wooden backing which is UV absorbing. The Plexiglas allowed maximum transmission of full-spectrum light (300–700 nm). A transparent optical filter (Photonitech Pte. Ltd., Singapore, Singapore) was fitted over the end of one of these chambers, which blocked all wavelengths between 300 and 400 nm (UV−). The other was left untouched (UV+). This filter was swapped between chambers every fourth trial to alleviate any potential influence of the chambers' appearance. Likewise, the chambers were randomized for each trial to counter any potential lateral bias. To ensure selection by *P. labiata* was based on UV and not on which animal moved first, we stimulated *C. umbratica* to move continuously throughout the trial. We found *C. umbratica* moved consistently when in a confined space. This was achieved by sealing the chambers with a square piece of Plexiglas attached to a stick, which was pushed into the chamber until it almost touched the spider. Before *C. umbratica* were placed on the frame, a *P. labiata* individual was placed in a 2 cm deep circular depression carved into the stem of the Y-frame. The depression was covered to prevent any visual stimulus during setup. Upon removal of the cover, each *P. labiata* individual was given 20 min to make a selection. The majority of individuals, however, made a selection within 5 min. A successful trial ended with *P. labiata* individuals striking at a *C. umbratica* individual. Due to a limited number of choice subjects, *C. umbratica* individuals were occasionally reused, but the same matching was never used with the same *P. labiata* individual. The order in which the pairings were used was randomized. Full-spectrum lighting conditions during the trials were as described in Lim et al. (2007a).

Experiment 1 examined whether *P. labiata* use the UV reflection of *C. umbratica* males as a prey cue. *Portia labiata* individuals (*n* = 10) were given the task of choosing between two live male *C. umbratica* (*n* = 32) with (UV+) and without (UV−) UV reflectance. All *P. labiata* individuals were subjected to 5 trials each with trial order randomized. *Cosmophasis umbratica* were weighed prior to the trials to 0.00001 g, and this weight was used as an indicator of size. Individuals then were size matched to the nearest 0.0001 g with the average size difference 0.00078 ± 0.00071 g.

Experiment 2 examined whether the display of sexually selected UV reflectance by male *C. umbratica* increases the risk of the males being consumed over the nonreflecting females. Individual *P. labiata* (*n* = 10) were presented with a choice between a male (*n* = 7) and a female (*n* = 7) *C. umbratica*. Each *P. labiata* was used in two repeated trials with the positioning of males and females (i.e., left or right) alternated each time.

Experiment 3 examined whether UV reflection is the sole cue used by *P. labiata* in selecting *C. umbratica* individuals as prey. In this experiment, UV reflectance of both the male and female choice subjects was masked. We had an a priori prediction that shows if the UV reflectance was the sole cue, then the choice made by *P. labiata* would be random. This experiment followed the same protocols of Experiment 2 but with both chambers of the Y-frame masked by UV-absorbing optical filters. Individuals used for Experiments 2 and 3 were different to individuals used for Experiment 1.

### Data analysis

All statistical tests were performed in R version 3.0.2 (R Development Core Team 2013). For all experiments, the dependent variable was binary representing the spiders' choice between UV+ and UV− treatments. For Experiment 1, the *Portia labiata* choice tests were analyzed using a generalized linear mixed effects model fitted with a logit-link function using the R-package *lme4* (Bates et al. 2014). *Portia labiata* individual ID was treated as a random factor to account for a lack of independence from the repeated measures design. The models examined whether the decision of *P. labiata* to attack a particular male *C. umbratica* was driven by the presence or absence of UV reflection. The difference in size, between the *C. umbratica* chosen by *P. labiata* and the individual not chosen, was explored as a covariate. This relative size index was mean centered and then divided by its standard error to ensure the variable was on a relative scale to the binary variable of UV reflectance. Trial number was also included to determine whether the decision by *P. labiata* was consistent across all trials. For each fixed effect, the following was reported: coefficient (i.e., *ß = *effect size), its standard error, the 95% confidence intervals, and the results from log-likelihood ratio tests, which were calculated through comparing fully specified models with the variable of interest removed.

For Experiments 2 and 3, *P. labiata* individuals were tasked with making a selection in two repeated trials. Despite the repeated measure design, a mixed model approach was not possible for either experiment due to quasicomplete separation. This stemmed from a high correlation between the binary response and explanatory variables. The data from experiment 2 and 3 were analyzed using Firth's logistic regression with the R-package *logistf* (Heinze et al. 2013), a method that corrects for such statistical issues. Statistical models for all experiments were simplified by backward elimination using a log-likelihood ratio test as criteria for variable removal. Variables with nonsignificant p-values from log-likelihood ratio tests were discarded with interaction terms tested and excluded first.

## Results

During trials, *P. labiata* actively assessed both subjects as evidenced by repeatedly rotating toward both choice subjects. In Experiment 1, *P. labiata* lunged at UV+ *C. umbratica* males for 43 of the 50 trials. This represented a significantly strong bias toward the UV stimulus (Table1) with a predicted probability of 86.2% that *P. labiata* will select a UV-reflecting male. The variance for the random effect of individual identity converged on zero suggesting the preference for UV-reflecting males was shared equally across all of the *P. labiata* sampled. In contrast, neither the relative size difference between choice subjects nor the trial number significantly influenced the outcome of the trials (Table1). When comparing the UV-reflecting males with the nonreflecting females, the trend was the same with *P. labiata* more frequently selecting the male (probability of selecting male: 87.5%; Table1). Lastly, this trend continued with *P. labiata* choosing males even though both males and females were masked with a UV filter (Probability of selecting male: 95%; Table1). For both these latter experiments, the observed responses were also independent of any potential influence of a difference in size or trial number (Table1).

**Table 1:** Generalized linear mixed effect model output for Experiment 1 and Firth's logistic regression output for experiments 2 and 3. Statistics include both variables excluded via backward elimination using log-likelihood ratio tests as criteria for elimination. The final model represents the variables of most importance. Bold *P*-values are significant at *P*< 0.05

	Coef	LCI (95%)	UCI (95%)	*χ*^2^	*P*-value
Experiment 1 – Comparing UV-reflecting males with UV-masked males					
Excluded variables					
Size difference: UV-reflecting male	0.506	−1.612	2.624	0.229	0.632
Trial				2.186	0.702
Trial 2	0.335	−2.299	2.969		
Trial 3	1.679	−1.004	4.362		
Trial 4	1.346	−1.333	4.025		
Trial 5	0.910	−1.648	3.468		
Size difference	−0.067	−0.908	0.773	0.025	0.875
Best model					
Intercept	−1.792	−3.014	−0.570		
UV-reflecting male	3.624	2.009	5.239	28.099	**1.15 × e-07**
Experiment 2 – Comparing UV-reflecting male with non-UV-reflecting females					
Excluded variables					
Size difference: UV-reflecting male	−0.903	−6.164	1.479	0.495	0.482
Trial	−1.215	−3.720	0.797	1.364	0.243
Size difference	0.357	−0.627	1.455	0.526	0.468
Best model					
Intercept	−0.201	−1.514	1.059	0.100	0.752
UV-reflecting male	2.147	0.199	4.642	4.716	**0.030**
Experiment 3 – Comparing UV-masked males with UV-masked females					
Excluded variables					
Size difference: Sex	0.191	−11.667	7.394	0.006	0.937
Trial	−0.487	−3.224	1.924	0.157	0.692
Size difference	0.698	−0.220	4.119	2.061	0.151
Best model					
Intercept	−0.511	−1.773	0.633	0.758	0.384
Sex	3.455	1.030	8.404	8.993	**0.003**

## Discussion

Our findings infer that the sexually selected UV signals of male *Cosmophasis umbratica* are used as a prey cue by the UV-sensitive predator *Portia labiata*. Consequently, by displaying these UV-mating signals, *C. umbratica* may be accruing potential fitness costs through an increased risk of predator detection. In contrast, the non-UV-reflecting females were rarely selected when paired with UV-reflecting males, which further supports the notion that UV signals are costly. No effect of a difference in size, between choice subjects, was recorded and indeed was not expected given the attempt at size matching individuals. Lastly, individuals were highly consistent in their selection as indicated by no influence of trial.

*Portia labiata* preferring males exhibiting UV reflection concurs with past findings that showed *P. labiata* preferentially selecting UV-reflective spiderwebs (Li and Lim 2005; Zou et al. 2011); a response shown to be independent of signal brightness. Just why *P. labiata* should choose UV-reflecting males over non-UV-reflecting males, however, is unclear. Female *C. umbratica* are known to assess males via the male-specific UV signals (Lim et al. 2007a), suggesting UV reflectance is linked to mate quality. It is thus tempting to suggest *P. labiata* similarly uses UV reflectance to assess prey quality. The intensity of the abdominal UV-green iridescence is greater in well-fed male *C. umbratica* then starved individuals (Lim and Li 2007). Hence, individuals with a more intense UV signal are likely to represent a better meal than males with less reflectance. This degree of fine-scale assessment is not unprecedented in spiders. Crab spiders (*Thomisus spectabilis*) for instance, use the same flower quality cues as honeybees when selecting a suitable flower for an ambush (Heiling and Herberstein 2004). Jumping spiders have high visual acuity and can learn to distinguish between colors for navigation to refuge sites (Hoefler and Jakob 2006) to avoid harmful stimulation (Nakamura and Yamashita 2000) and to discriminate between prey (Taylor et al. 2014). It is possible then that *P. labiata* can use UV cues to detect and discriminate between specific preys or even make fine-scale assessments about intraspecific prey quality. However, to our knowledge, this has not been tested for any system to date.

The likelihood of *P. labiata* having to choose between two or more male *C. umbratica* in the field, however, is probably rare. Instead, the capacity to detect UV is more likely to simply aid with the mechanics of prey capture. Foliage rarely reflects bright UV, so for a UV-sensitive predator, brightly UV-reflecting objects are generally easier to discriminate from their backgrounds (Honkavaara et al. 2002). Easier detection of prey objects should select for sensitivity to UV reflection. UV reflectance is also effective in low light conditions (Olofsson et al. 2010) making it a versatile prey cue in a variety of light conditions. This is potentially very important given *P. labiata*, like other species, is found in tropical rainforests where light levels are highly variable (Jackson and Pollard 1996). UV reflectance could also provide additional visual information that aids perception of depth, which is necessary for *P. labiata*'s lunging attack. The green-sensitive visual pigment in the eye of jumping spiders is used in this process (Nagata et al. 2012, 2013), but the role of UV pigments has yet to be investigated.

It is clear from our findings that UV signals are not the only cue(s) stimulating *P. labiata* to attack male *C. umbratica*. *Portia labiata* still exhibited a strong preference toward male *C. umbratica* over females when UV reflection was masked for both sexes. This result may have a variety of interpretations such as: the UV markings co-vary with some other male-specific traits that provide equal detectability or; males are more regularly encountered than females and hence are more recognizable as a prey species or; females are considered more dangerous than the males and are hence avoided. Either way it suggests *P. labiata* still exploit male-specific traits of *C. umbratica* in the absence of UV cues. Jumping spiders in general, and *Portia* in particular, are capable of utilizing a variety of cues when selecting a mate (Taylor and McGraw 2013) or hunting prey (Harland and Jackson 2000). In a sense, this is paramount in circumstances where variable environmental conditions render some cues ineffective (Taylor and McGraw 2013). Coloration is not the only feature that differs between the sexes of *C. umbratica*. Males have substantial palps, a more slender abdomen and longer legs than the females (Lim and Li 2004). *Portia,* in general, are capable of discriminating between such morphological traits (Harland and Jackson 2000). Male jumping spiders in general also tend to be more active than females and will roam further afield (Hoefler and Jakob 2006). Hence, *P. labiata* potentially encounter males more often, and so male *C. umbratica* may simply be a more familiar prey item for *P. labiata*.

Our study indicates that *P. labiata* exploits the male-specific UV signals of *C. umbratica*. It suggests UV-sensitive predators, such as *P. labiata*, may collectively moderate the conspicuousness of sexually selected UV markings in male *C. umbratica*. Irrespective of the information content of UV cues, *P. labiata* clearly presented a preference for UV-reflective males over non-UV-reflecting males. However, it appears UV reflectance is not the only male-specific cue used by *P. labiata*. Further investigations are required to better understand how UV coloration and the predator–prey relationship between *P. labiata* and *C. umbratica* play out under natural conditions.

## References

[b1] Andersson MB (1994). Sexual selection.

[b2] Bates D, Maechler M, Bolker B, Walker S (2014). http://CRAN.R-project.org/package<lme4.

[b3] Bernal XE, Akre KL, Baugh AT, Rand AS, Ryan MJ (2006). Acoustic preferences and localization performance of blood-sucking flies (*Corethrella* Coquillett) to túngara frog calls. Behav. Ecol.

[b4] Elias DO, Land BR, Mason AC, Hoy RR (2006). Measuring and quantifying dynamic visual signals in jumping spiders. J. Comp. Physiol. A Neuroethol. Sens. Neural. Behav. Physiol.

[b5] Girard MB, Endler JA (2014). Peacock spiders. Curr. Biol.

[b6] Harland DP, Jackson RR (2000). Cues by which *Portia fimbriata*, an araneophagic jumping spider, distinguishes jumping-spider prey from other prey. J. Exp. Biol.

[b7] Harland DP, Li D, Lazareva FO, Shimizu T, Wasserman EA, Jackson RR (2012). How jumping spider see the world. How animals see the world.

[b8] Heiling AM, Herberstein ME (2004). Floral quality signals lure pollinators and their predators. Ann. Zool. Fenn.

[b9] Heinze G, Ploner M, Dunkler D, Southworth H (2013).

[b10] Hoefler CD, Jakob EM (2006). Jumping spiders in space: movement patterns, nest site fidelity and the use of beacons. Anim. Behav.

[b11] Honkavaara J, Koivula M, Korpimäki E, Siitari H, Viitala J (2002). Ultraviolet vision and foraging in terrestrial vertebrates. Oikos.

[b12] Hu Z, Liu F, Xu X, Chen Z, Chen J, Li D (2012). Spectral transmission of the principal-eye corneas of jumping spiders: implications for ultraviolet vision. J. Exp. Biol.

[b13] Husak JF, Macedonia JM, Fox SF, Sauceda RC (2006). Predation cost of conspicuous male coloration in collared lizards (*Crotaphytus collaris*): an experimental test using clay-covered model lizards. Ethology.

[b14] Jackson RR, Hallas SEA (1986). Comparative biology of *Portia africana*
*P. albimana*
*P. fimbriata*
*P. labiata*, and *P. shultzi*, araneophagic, web-building jumping spiders (Araneae: Salticidae): Utilisation of webs, predatory versatility, and intraspecific interactions. N. Z. J. Zool.

[b15] Jackson R, Pollard S (1996). Predatory behaviour of jumping spiders. Annu. Rev. Entomol.

[b16] Kodric-Brown A (1985). Female preference and sexual selection for male coloration in the guppy (*Poecilia reticulata*. Behav. Ecol. Sociobiol.

[b17] Kuijper B, Pen I, Weissing FJ (2012). A Guide to Sexual Selection Theory. Annu. Rev. Ecol. Evol. Syst.

[b18] Li D, Lim MLM (2005). Ultraviolet cues affect the foraging behaviour of jumping spiders. Anim. Behav.

[b19] Lim ML, Li D (2004). Courtship and male–male agonistic behaviour of *Cosmophasis umbratica* Simon, an ornate jumping spider (Araneae: Salticidae) from Singapore. Raffles Bull. Zool.

[b20] Lim ML, Li D (2006). Extreme ultraviolet sexual dimorphism in jumping spiders (Araneae: Salticidae). Biol. J. Linn. Soc.

[b21] Lim MLM, Li D (2007). Effects of age and feeding history on structure-based UV ornaments of a jumping spider (Araneae: Salticidae). Proc. R. Soc. B Biol. Sci.

[b22] Lim MLM, Li D (2013). UV-green iridescence predicts male quality during jumping spider contests. PLoS ONE.

[b23] Lim MLM, Li J, Li D (2007a). Effect of UV-reflecting markings on female mate-choice decisions in *Cosmophasis umbratica*, a jumping spider from Singapore. Behav. Ecol.

[b24] Lim MLM, Land MF, Li D (2007b). Sex-specific UV and fluorescence signals in jumping spiders. Science.

[b25] Nagata T, Koyanagi M, Tsukamoto H, Saeki S, Isono K, Shichida Y (2012). Depth perception from image defocus in a jumping spider. Science.

[b26] Nagata T, Arikawa K, Terakita A (2013). Contribution of a visual pigment absorption spectrum to a visual function: depth perception in a jumping spider. Biophysics.

[b27] Nakamura T, Yamashita S (2000). Learning and discrimination of colored papers in jumping spiders (Araneae, Salticidae). J. Comp. Physiol. A Neuroethol. Sens. Neural. Behav. Physiol.

[b28] Olofsson M, Vallin A, Jakobsson S, Wiklund C (2010). Marginal eyespots on butterfly wings deflect bird attacks under low light intensities with UV wavelengths. PLoS ONE.

[b29] R Development Core Team (2013).

[b30] Stuart-Fox DM, Ord TJ (2004). Sexual selection, natural selection and the evolution of dimorphic coloration and ornamentation in agamid lizards. Proc. R. Soc. B Biol. Sci.

[b31] Stuart-Fox DM, Moussalli A, Marshall NJ, Owens IPF (2003). Conspicuous males suffer higher predation risk: visual modelling and experimental evidence from lizards. Anim. Behav.

[b32] Taylor LA, McGraw KJ (2013). Male ornamental coloration improves courtship success in a jumping spider, but only in the sun. Behav. Ecol.

[b33] Taylor LA, Maier EB, Byrne KJ, Amin Z, Morehouse NI (2014). Colour use by tiny predators: jumping spiders show colour biases during foraging. Anim. Behav.

[b34] Tovée MJ (1995). Ultra-violet photoreceptors in the animal kingdom: their distribution and function. Trends Ecol. Evol.

[b35] Zou Y, Araujo DP, Lim ML, Li D (2011). Ultraviolet is a more important cue than reflection in other wavelengths for a jumping spider to locate its spider prey. Anim. Behav.

